# Survey of clinical and commensal *Escherichia coli* from commercial broilers and turkeys, with emphasis on high-risk clones using APECTyper

**DOI:** 10.1016/j.psj.2023.102712

**Published:** 2023-04-13

**Authors:** Jodi Delago, Elizabeth A. Miller, Cristian Flores-Figueroa, Jeannette Munoz-Aguayo, Carol Cardona, Alexandra H. Smith, Timothy J. Johnson

**Affiliations:** ⁎ Arm and Hammer Animal and Food Production, Waukesha, WI, 53186, USA; † Department of Veterinary and Biomedical Sciences, University of Minnesota, St. Paul, MN, USA; ‡ Mid-Central Research and Outreach Center, University of Minnesota, Willmar, MN, USA

**Keywords:** poultry, *Escherichia coli*, APEC, colibacillosis, pathotype

## Abstract

Molecular characterization of avian pathogenic *Escherichia coli* (**APEC**) is challenging due to the complex nature of its associated disease, colibacillosis, in poultry. Numerous efforts have been made toward defining APEC, and it is becoming clear that certain clonal backgrounds are predictive of an avian *E. coli* isolate's virulence potential. Thus, APEC can be further differentiated as high-risk APEC based upon their clonal background's virulence potential. However, less clear is the degree of overlap between clinical isolates of differing bird type, and between clinical and gastrointestinal isolates. This study aimed to determine genomic similarities and differences between such populations, comparing commercial broiler vs. turkey isolates, and clinical vs. gastrointestinal isolates. Differences were observed in Clermont phylogenetic groups between isolate populations, with B2 as the dominant group in turkey clinical isolates and G as the dominant group in broiler clinical isolates. Nearly all clinical isolates were classified as APEC using a traditional gene-based typing scheme, whereas 53.4% and 44.1% of broiler and turkey gastrointestinal isolates were classified as APEC, respectively. High-risk APEC were identified among 31.0% and 46.9% of broiler and turkey clinical isolates, compared with 5.7% and 2.9% of broiler and turkey gastrointestinal isolates. As found in previous studies, no specific known virulence or fitness gene sets were identified which universally differentiate between clinical and gastrointestinal isolates. This study further demonstrates the utility of a hybrid APEC typing approach, considering both plasmid content and clonal background, for the identification of dominant and highly virulent APEC clones in poultry production.

## INTRODUCTION

Avian colibacillosis is a disease caused by *Escherichia coli*, and its impact on poultry production across the world is significant (Nolan et al., 2020). For this reason, a relatively large volume of research over the past decades has been focused on understanding why colibacillosis occurs, and how it may be mitigated. This research has been broadly aimed at 2 facets of the disease. First, research has attempted to understand the host and environmental factors which predispose the bird to colibacillosis. This work has determined that colibacillosis is often secondary in nature to some predisposing stress (Nolan et al., 2020). However, the second arm of research has been focused on the causative agent of the disease, *E. coli*. Much of this work has centered around identifying genetic traits which enhance disease-causing ability, and defining the *E. coli* causing disease by their possession of unique genomic markers (Dho-Moulin and Fairbrother, 1999).

The search for genomic markers which define avian *E. coli* causing colibacillosis have led to the proposal of a pathotype for this collective group referred to as avian pathogenic *E. coli*, or APEC (Collingwood et al., 2014). Much like other *E. coli* pathotypes, such as Enterotoxigenic *E. coli* (**ETEC**), the goal of establishing the APEC pathotype was to ultimately define a specific set of genomic markers indicate of this group. Unfortunately, this seemingly simple task has been very difficult to achieve. Like other extraintestinal *E. coli*, APEC do not possess a universal subset of genes which make them unique from other *E. coli* (Dale and Woodford, 2015). The reasons for this, in part, lie in the nature of the disease. First, the gastrointestinal tract of the bird is a reservoir for APEC, and APEC residing in the gut do not cause disease (Kemmett et al., 2013). This creates a problem distinguishing clinical isolates from true fecal, gastrointestinal, or environmental isolates. Second, because colibacillosis is often a secondary disease following a primary stressor in the bird, the amount of stress induced correlates with a strain's ability to induce disease. That is, under mild to moderate stress, only *E. coli* strains with enhanced virulence potential are likely able to cause colibacillosis. However, when severe stress is induced, it is likely that most if not all *E. coli* strains have the potential to cause disease (Collingwood et al., 2014). Because of this, clinical isolates are confounded and contain a mixture of highly virulent APEC plus generic *E. coli* that were in the right place, at the right time.

Despite these challenges in defining APEC, progress has been made toward defining the pathotype. Early on, it was realized that a common trait of clinical *E. coli* was the possession of a large plasmid encoding microcins (Wooley et al., 1993). This plasmid, referred to as the “ColV plasmid” or later “APEC plasmid,” has repeatedly been found at high prevalence across the world in clinical isolate populations. The first completed genome sequence of a ColV plasmid identified a conserved island of virulence and fitness factors carried by the plasmid (Johnson et al., 2006b), and subsequent work presented a typing scheme that can be used to define APEC based on possession of conserved genes of this island (Johnson et al., 2008), which were found significantly less often in fecal isolates from healthy birds (Rodriguez-Siek et al., 2005). Other key work has been performed to identify additional chromosomal virulence factors which contribute to APEC's ability to cause disease (Dozois et al., 2003; Ewers et al., 2004). Some of these genes have been incorporated into a second commonly used typing scheme (Ewers et al., 2005). These genes are infrequently present among APEC, but might aid in the identification of APEC with enhanced virulence potential (Rodriguez-Siek et al., 2005).

Recently, genomic-based research was presented illustrating an additional problem with defining the APEC pathotype: genes previously used to differentiate between APEC and avian commensal *E. coli* are now found at much higher prevalence in the commensal populations (Mageiros et al., 2021). Based upon this observation, we conducted a genomics-based study examining more than 3,000 isolates from clinical disease in commercial turkeys, and compared these isolates with cecal isolates from apparently healthy birds at slaughter (Johnson et al., 2022). Congruent with recent work, previously identified APEC virulence and fitness factors were found at high prevalence in the cecal isolate populations. However, we determined that the majority of clinical isolates belong to one of 5 sequence types: ST23, ST117, ST131, ST355, and ST428. These STs were absent or found at very low prevalence among cecal isolates. Furthermore, a virulence model in avian embryos showed that isolates from these 5 STs were highly lethal, irrespective of APEC virulence factor carriage, and cecal isolates from dominant cecal STs were avirulent, irrespective of APEC virulence factor carriage. This prompted us to develop a new scheme for the identification of high-risk APEC, called APECTyper (Johnson et al., 2022).

A limitation of our previous work was the exclusion of clinical isolates from broilers. A second limitation was that we examined cecal isolates at slaughter, and not isolates from other areas of the gastrointestinal tract taken on farm. Thus, the purpose of the present study was to characterize isolates from such populations, and determine if the high-risk APEC typing scheme is valid when considering commercial broiler isolate populations.

## MATERIALS AND METHODS

### Collection of Isolates

This work was reviewed by the University of Minnesota Institutional Animal Care and Use Committee, and deemed to be exempt from a need for protocol approval. A total of 180 samples of convenience were collected from commercial broiler and turkey operations. These included samples from broilers with colibacillosis (*n* = 58), the gastrointestinal tracts of healthy broilers (*n* = 88), and the gastrointestinal tracts of healthy turkeys (*n* = 34). Clinical isolates from broilers were collected from birds displaying classical signs of colibacillosis (air sacculitis, pericarditis, perihepatitis). Swabs from internal organs of these birds were streaked onto MacConkey agar (BD Difco, Franklin Lakes, NJ) and incubated overnight at 37°C. Nonclinical isolates were collected from the small intestinal mucosa of presumably healthy broilers (free from apparent lesions indicating disease). From these birds, 6 cm sections of the duodenum, ileum, and jejunum were dissected, rinsed with sterile 0.1% peptone (Gibco, Detroit, MI), diluted 1:10 with sterile 0.1% peptone, masticated at 300 rpm for 1 min in a stomacher (Model 400 circulator, Seward, England) to dislodge the mucosal layer, serially diluted and pour-plated on CHROMagar ECC (CHROMagar, Paris, France), and incubated overnight at 37°C. For comparison purposes, we also included 397 clinical isolates from commercial turkeys, which were collected in a similar manner to the clinical broiler isolates described above (Johnson et al., 2022).

Following incubation, one suspect *E. coli* colony was used from each gastrointestinal tract sample for this study. Similarly, only one sample was used from a barn experiencing colibacillosis-associated deaths at a given timepoint. Samples specific to this study were collected between July 2014 and January 2020, with 180 total isolates collected (Supplementary Data). Isolates were collected from 28 poultry producing companies in the United States, across at least 13 different states and 111 different farms. Ages ranged from 7 to 74 days. All isolates were later confirmed to be *E. coli* through DNA sequencing (see below).

### DNA Extraction and Sequencing

All isolates were sequenced in this study using Illumina short-read technology. DNA was extracted from overnight TS broth (BD Difco) cultures of a single colony using the DNeasy Blood & Tissue kit (Qiagen, Valencia, CA) following the manufacturer's instructions. Genomic DNA libraries were created using the Nextera XT DNA library preparation kit and Nextera XT index kit v3 (Illumina, San Diego, CA), and sequencing was performed using 2 × 300-bp dual-index runs on an Illumina MiSeq.

### Genome Assembly and Quality Assessment

Raw FASTQ files for each genome were trimmed and quality filtered using Trimmomatic (v0.33) (Bolger et al., 2014), including removal of Illumina adapters, with a sliding window of 4 and average Phred quality score of 20, and 36 as the minimum read length. Assemblies of each genome were performed using Shovill (v1.0.4), specifying the SPAdes assembler (Bankevich et al., 2012), with default parameters (https://github.com/tseemann/shovill). Assembly quality was assessed with QUAST (v5.0.0) (Gurevich et al., 2013).

### Serotype and Sequence Type Prediction

In silico serotype prediction was performed with ECTyper (v1.0.2) (Bessonov et al., 2021) using a minimum sequence identity of 50% and minimum hit coverage of 50%. Serotypes were reported as O and H antigen types. In silico multilocus sequence typing (MLST) was performed using mlst (v2.16.1) (https://github.com/tseemann/mlst), using the 7-gene *E. coli* MLST scheme hosted on the PubMLST website (https://pubmlst.org) (Jolley and Maiden, 2010).

### Genetic Feature Identification

ABRicate (v.0.8.13) (https://github.com/tseemann/abricate) was used with a minimum identity of 90% and minimum coverage of 80% to screen isolate genome assemblies for *E. coli* virulence factors (Malberg Tetzschner et al., 2020), acquired antimicrobial resistance genes (Zankari et al., 2012), and plasmid replicons (Carattoli et al., 2014). EZClermont was used to predict *E. coli* phylogenetic group for each isolates using default parameters (Waters et al., 2020). A custom APEC database consisting of 46 genes was created using a scan of existing literature for genes linked to APEC virulence or fitness through direct evidence or epidemiological association, as previously described (Johnson et al., 2022). ABRicate was used with a minimum identity of 90% and minimum coverage of 80% to determine APEC gene prevalence across all isolates. APECtyper was used to determine if isolates belonged to a “high-risk” clonal group, as previously described (Johnson et al., 2022). Heatmaps were constructed with ClustVis using average clustering (https://biit.cs.ut.ee/clustvis/).

### Phylogenetic Analyses

Single nucleotide polymorphisms (**SNPs**) were identified in each sample using Snippy (v4.4.0), with a minimum sequencing depth of 8× (https://github.com/tseemann/snippy) and *E. coli* strain APEC 078 used as the reference (Mangiamele et al., 2013). A core SNP alignment was then created for all isolates (*n* = 576), containing 83,128 informative sites. A maximum likelihood tree was then reconstructed with IQ-TREE (v1.6.10), using 1,000 ultrafast bootstrap iterations (Nguyen et al., 2015a). ModelFinder was used to identify the most appropriate substitution model (Kalyaanamoorthy et al., 2017). For the tree, GTR+F+ASC+R7 was used. The Interactive Tree of Life was used for tree construction (Letunic and Bork, 2016).

### Statistical Analyses

For distributions of resistance genes, APEC genes, *E. coli* virulence factors, and plasmid replicons, a chi-square test of probability was performed to determine if distributions differed between groups examined.

### Data Availability

Raw reads from isolates sequenced in this study are available at the NCBI Short Read Archive (**SRA**) under BioProject accession no. PRJNA799011. APECtyper is available at: https://github.com/JohnsonSingerLab/APECtyper. The APEC virulence gene database is available at: https://github.com/JohnsonSingerLab/APEC_VF_database.

## RESULTS AND DISUSSION

### Phylogenetic Groups

Among broiler clinical isolates, the dominant Clermont phylogenetic group was G (46.4%) (Table 1). This was significantly higher prevalence of group G (*P* < 0.05) than any other source population examined. Remaining broiler clinical isolates were distributed across the B1, B2, C, D, and E groups. In contrast, the dominant phylogenetic groups in broiler gastrointestinal isolates were A and B1 (52.2% total), with remaining isolates distributed across all other phylogenetic groups. Turkey clinical isolates differed from broiler clinical isolates in their distribution of phylogenetic groups. The dominant turkey clinical group was B2 (45.6%), which was significantly higher among this population than all others examined (*P* < 0.05). Turkey clinical isolates also had significantly higher proportions of isolates belonging to the C phylogenetic group (18.6%) than other populations examined (*P* < 0.05). In contrast, turkey gastrointestinal isolates were most frequently identified as belonging to the B1 phylogenetic group (26.5%), and other isolates were distributed across all other phylogenetic groups except C.

**Table 1:** Distribution of Clermont phylogenetic groups (%) among *E. coli* populations from broilers and turkeys.

Clermont phylotype	A	B1	B2	C	D	E	F	G
Broiler clinical (*n* = 58)	0.0^b^	16.1^a^	12.5^b^	5.4^b^	10.7	7.1^b^	0.0	46.4^a^
Broiler gastrointestinal (*n* = 88)	27.8^a^	24.4^a^	5.6^b^	3.3^b^	15.6	6.7^b^	3.3	11.1^b^
Turkey clinical (*n* = 397)	1.8^b^	7.8^b^	45.6^a^	18.6^a^	11.3	2.0^c^	3.0	9.6^b^
Turkey gastrointestinal (*n* = 34)	5.9^b^	26.5^a^	8.8^b^	0.0^b^	17.6	14.7^a^	5.9	14.7^b^
*P* value	<0.01	<0.01	<0.01	<0.01	0.52	<0.01	0.43	<0.01

a-c Indicate differences between groups based on statistical tests (P < 0.05).

### Sequence Types

The overall dominant sequence types identified in this study included ST117 (51.6% of total), ST131 (24.9%), ST23 (20.2%), ST428 (12.6%), and ST349 (12.4%), although numerous sequence types were identified and many of these were *n* = 1 or *n* = 2 in count (Table 2). For broiler clinical isolates, the most prevalent STs included ST117, ST131, ST101, ST602, ST95, and ST5940. ST117 dominated the broiler clinical landscape, with 28.6% of isolates belonging to this ST. Interestingly, only 37.5% of these ST117 isolates were predicted to contain the O78 serogroup. We had previously determined that the virulence potential for O78-ST117 isolates is higher than ST117 isolates belonging to other serogroups, and virulence was quite variable within ST117 (Johnson et al., 2022). However, a limited number of serogroups were tested in that study (O78, O109, O111, and O119), and ST117 contains strains belonging to a large number of different serogroups. Also, some of these serogroups (O109 and O111) were still classified as virulent, even though they were considerably less lethal than O78 isolates. None of the other serogroups previously examined from turkey-source isolates (O109, O111, and O119) were present in broiler-source isolates in this study, but other ST117 serogroups were, including O11, O71, and O161. Based on its global dominance (Mora et al., 2012; Ronco et al., 2017; Mageiros et al., 2021; Mehat et al., 2021), it is likely that serogroups of ST117 other than O78 are of higher risk, and thus in commercial broilers all ST117 should likely be considered potentially high risk. Turkey clinical isolates have been examined in a previous study, and dominant STs included ST23, ST131, ST428, ST117, and ST355 (Johnson et al., 2022).

**Table 2:** Distribution of dominant sequence types (%) among *E. coli* populations from broilers and turkeys.

Sequence type	Broiler clinical (*n* = 58)	Broiler gastrointestinal (*n* = 88)	Turkey clinical (*n* = 397)	Turkey gastrointestinal (*n* = 34)	Percent overall
117	28.6	5.6	8.6	8.8	51.6
131	7.1	2.2	12.6	2.9	24.9
23	3.6	2.2	14.4	0.0	20.2
428	0.0	0.0	12.6	0.0	12.6
349	3.6	1.1	4.8	2.9	12.4
4197	0.0	0.0	0.0	11.8	11.8
501	0.0	5.6	0.0	5.9	11.4
10	0.0	10.0	0.3	0.0	10.3
101	7.1	2.2	0.3	0.0	9.7
155	0.0	8.9	0.5	0.0	9.4
602	5.4	3.3	0.0	0.0	8.7
58	0.0	1.1	1.5	2.9	5.6
355	0.0	0.0	5.5	0.0	5.5
95	5.4	0.0	0.0	0.0	5.4
5940	5.4	0.0	0.0	0.0	5.4
57	3.6	1.1	0.0	0.0	4.7
2538	0.0	4.4	0.0	0.0	4.4
2792	1.8	2.2	0.3	0.0	4.3
134	0.0	3.3	0.0	0.0	3.3
43	0.0	3.3	0.0	0.0	3.3
38	0.0	2.2	1.0	0.0	3.2
752	0.0	2.2	0.0	0.0	2.2

Broiler gastrointestinal isolates were widely distributed across STs, and the most commonly isolated STs included ST10, ST155, ST117, and ST501.The most common turkey gastrointestinal isolates included ST4197, ST117, and ST501. Both of these results differ somewhat from our previous cecal isolate data (Johnson et al., 2022). In that study, broiler cecal dominant STs included ST189, ST10, ST117, and ST155. Dominant turkey cecal STs included ST58, ST10, ST155, and ST69. This suggests that 1) gastrointestinal and cecal isolates from broiler and turkeys might innately differ in their distribution of clones for unexplained reasons, and 2) there seems to be distinctions between clones colonizing the ceca of the bird vs. those colonizing the main intestinal tract. Both of these observations warrant further study.

### High-Risk Clones

Using APECTyper, which identifies high-risk APEC clones using a combination of searches for serogroup, sequence type, and APEC plasmid genes, 96.6% of broiler clinical isolates and 91.2% of turkey clinical isolates were identified as APEC, defined via possession of *hlyF* and *ompTp* of the APEC plasmid (Table 3). In contrast, only 46.6% of broiler gastrointestinal and 55.9% of turkey gastrointestinal isolates were classified as APEC, respectively. Using APECTyper's definition of a high-risk clone (*hlyF* and *ompTp* in combination with either O78, ST117-O78, ST131, ST23, ST355, ST428), 31.0% of broiler clinical isolates and 46.9% of turkey clinical isolates were identified as high risk. In contrast, high-risk APEC were only identified among 5.7% and 2.9% of broiler and turkey gastrointestinal isolates, respectively. Because ST117 has been recognized as a globally dominant broiler ST but its presence in an isolate alone is not enough to classify an APEC as high-risk (the O78 serogroup must be found in combination with ST117), we also examined high-risk clones plus any ST117 isolates among the populations. Using these criteria, 48.3% of broiler clinical isolates and 55.4% of turkey isolates were identified as high risk, compared to 11.4% and 11.8% of broiler and turkey gastrointestinal isolates, respectively. These data demonstrate the ability of APECTyper to differentiate clinical vs. gastrointestinal isolates with high virulence potential. They also illustrate that between one third and one half of clinical isolates possess high-risk potential, and that these clones are found infrequently in the gut.

**Table 3:** Classification of *E. coli* isolates from broilers and turkeys using traditional APEC typing (APEC) and APECTyper (high-risk APEC).

	Non-APEC (%)	APEC (%)	High-risk APEC (%)	High-risk APEC including all ST117 (%)
Broiler clinical (*n* = 58)	3.4	96.6	31.0	48.3
Broiler gastrointestinal (*n* = 88)	53.4	46.6	5.7	11.4
Turkey clinical (*n* = 397)	8.8	91.2	46.9	55.4
Turkey gastrointestinal (*n* = 34)	44.1	55.9	2.9	11.8

### Virulence and Fitness Factors

**Figure 1:**
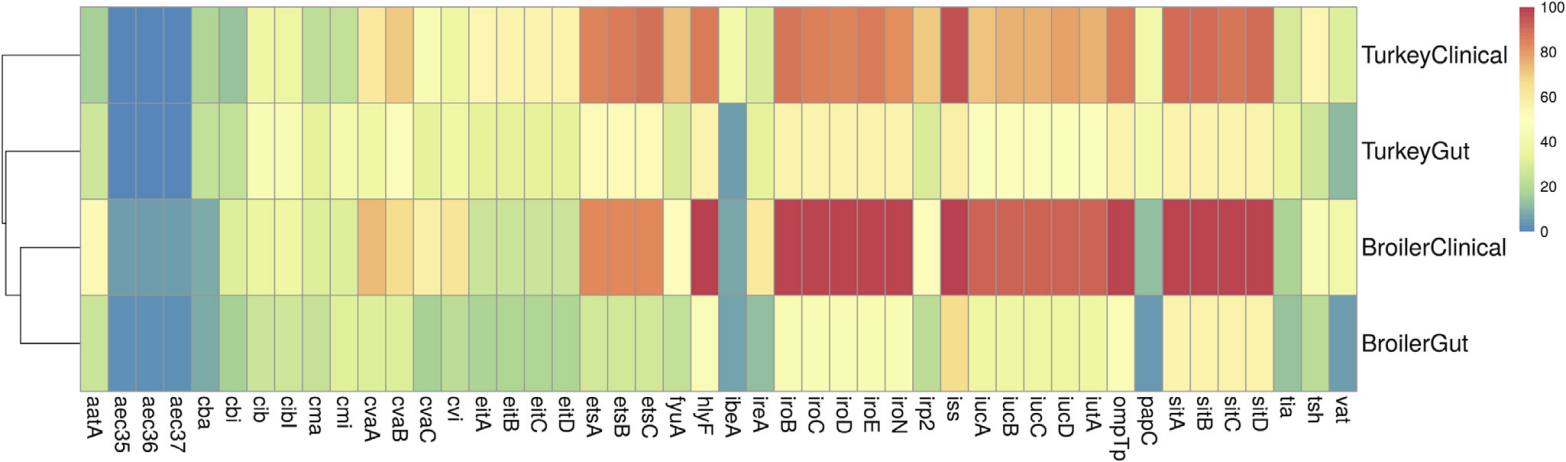
Heatmap depicting the prevalence of known APEC virulence and fitness factors across clinical and gastrointestinal isolates from broilers and turkeys. Colors represent percent presence of a gene within a source group.

Two databases were searched for the presence of known virulence and fitness factors among the isolates within this study. First, the presence of APEC virulence-associated genes was examined across populations using a custom APEC database containing 46 different genes. Of these, 42/46 genes significantly differed in their prevalence (*P* < 0.05) across the populations studied (Table 4 and Figure 1). A number of genes were significantly more prevalent in the broiler clinical population compared to the broiler gastrointestinal population (*P* < 0.05), including *aatA, aec35-37, cvaA-C, etsA-C, fyuA, hlyF, ireA, iroB-N, irp2, iss, iucA-D, iutA, ompTp, papC, sitA-D, tsh,* and *vat*. A number of these genes were identified in >80% of broiler clinical isolates, including *etsA-C, hlyF, iroB-N, iss, iucA-D, ompTp*, and *sitA-D*. All of these genes have been previously localized to the conserved regions of the APEC plasmid (Johnson et al., 2006a,b). Among broiler gastrointestinal isolates, the prevalence of these genes ranged from 27.8% to 66.7%, and among turkey gastrointestinal isolates they ranged from 47.1% to 58.8%. These results further support the widespread conservation of virulence and fitness genes of the APEC plasmid across clinical isolates from broilers and turkeys. It also validates that these same genes are more prevalent among gastrointestinal isolates from healthy broilers and turkeys than previously realized, underscoring the need for better typing systems to differentiate APEC of differing virulence capacities.

**Table 4:** Distribution of known avian pathogenic *E. coli* virulence and fitness factors (%) among *E. coli* populations from broilers and turkeys.

	Broiler clinical (*n* = 58)	Broiler gastrointestinal (*n* = 88)	Turkey clinical (*n* = 397)	Turkey gastrointestinal (*n* = 34)	Chi-squared *P* value
*iss*	100.0^a^	66.7^b^	96.0^a^	58.8^b^	<0.01
*sitA*	98.2^a^	55.6^c^	89.9^b^	55.9^c^	<0.01
*sitB*	98.2^a^	56.7^c^	89.9^b^	55.9^c^	<0.01
*sitD*	98.2^a^	56.7^c^	89.9^b^	55.9^c^	<0.01
*hlyF*	100.0^a^	45.6^c^	86.4^b^	55.9^c^	<0.01
*iroB*	98.2^a^	44.4^c^	87.4^b^	55.9^c^	<0.01
*sitC*	98.2^a^	56.7^c^	87.4^b^	55.9^c^	<0.01
*ompTp*	98.2^a^	46.7^c^	86.6^b^	55.9^c^	<0.01
*iroE*	98.2^a^	44.4^c^	86.1^b^	55.9^c^	<0.01
*iroC*	98.2^a^	44.4^c^	85.6^b^	55.9^c^	<0.01
*iroD*	98.2^a^	44.4^c^	84.9^b^	55.9^c^	<0.01
*iroN*	98.2^a^	44.4^c^	82.9^b^	55.9^c^	<0.01
*etsC*	83.9^a^	27.8^c^	88.7^a^	52.9^b^	<0.01
*etsB*	83.9^a^	27.8^c^	86.9^a^	52.9^b^	<0.01
*iucD*	91.1^a^	36.7^c^	78.3^b^	47.1^c^	<0.01
*etsA*	83.9^a^	27.8^c^	84.9^a^	52.9^b^	<0.01
*iucB*	91.1^a^	37.8^c^	75.8^b^	47.1^c^	<0.01
*iutA*	91.1^a^	37.8^c^	75.8^b^	47.1^c^	<0.01
*iucC*	91.1^a^	36.7^c^	75.1^b^	47.1^c^	<0.01
*iucA*	91.1^a^	37.8^c^	72.0^b^	47.1^c^	<0.01
*cvaB*	66.1^a^	30.0^b^	70.5^a^	47.1^b^	<0.01
*cvaA*	73.2^a^	30.0^b^	61.0^a^	38.2^b^	<0.01
*fyuA*	51.8^b^	23.3^c^	72.3^a^	29.4^c^	<0.01
*irp2*	51.8^b^	21.1^c^	70.8^a^	29.4^c^	<0.01
*cvaC*	58.9^a^	16.7^c^	44.8^b^	32.4^bc^	<0.01
*cvi*	62.5^a^	21.1^c^	37.0^b^	38.2^bc^	<0.01
*tsh*	44.6^ab^	21.1^c^	54.4^a^	26.5b^c^	<0.01
*ireA*	60.7^a^	12.2^c^	29.5^b^	32.4^b^	<0.01
*eitD*	25.0^b^	18.9^b^	56.2^a^	32.4^b^	<0.01
*eitB*	25.0^b^	18.9^b^	55.2^a^	32.4^b^	<0.01
*eitC*	25.0^b^	18.9^b^	55.2^a^	32.4^b^	<0.01
*eitA*	25.0^b^	17.8^b^	54.9^a^	32.4^b^	<0.01
*cibI*	37.5	26.7	36.3	44.1	0.22
*cib*	35.7	25.6	37.0	44.1	0.15
*aatA*	53.6^a^	25.6^b^	16.9^b^	26.5^b^	<0.01
*vat*	39.3^a^	5.6^b^	30.7^a^	11.8^b^	<0.01
*cmi*	30.4	31.1	23.9	41.2	0.09
*cma*	30.4	24.4	22.9	32.4	0.44
*papC*	12.5^b^	4.4^b^	39.0^a^	41.2^a^	<0.01
*ibeA*	7.1^b^	6.7^b^	39.8^a^	5.9^b^	<0.01
*tia*	17.9^ab^	13.3^b^	28.2^a^	35.3^a^	<0.01
*cbi*	30.4^a^	16.7^ab^	13.6^b^	23.5^ab^	<0.01
*cba*	8.9^ab^	8.9^b^	18.9^a^	23.5^a^	0.03
*aec35*	5.4^a^	2.2^b^	0.3^b^	0.0^bc^	<0.01
*aec36*	5.4^a^	2.2^b^	0.3^b^	0.0^bc^	<0.01
*aec37*	5.4^a^	2.2^b^	0.3^b^	0.0^bc^	<0.01

a-c Indicate differences between groups based on statistical tests (P < 0.05).

The same data were also compared when isolates were categorized as non-APEC, non–high-risk APEC, or high-risk APEC according to APECTyper. Here, statistical comparison was performed between high-risk APEC vs. non–high-risk APEC, and several genes were identified which were of significantly different prevalence (*P* < 0.05) (Table 5). Genes which were significantly higher in high-risk APEC included *cvaB, eitABCD, fyuA, ibeA, irp2, iucABCD, iutA, papC, tia*, and *tsh*. Of these genes, *cvaB, eitABCD, iucABCD, iutA*, and *tsh* are typically located on ColV- or ColBM-type APEC plasmids (Johnson et al., 2006a,b). Typical chromosomally encoded genes include *fyuA, ibeA, irp2, papC*, and *tia*. Certainly, different variants of the APEC plasmids exist, and these results prompt the possibility that possession of different plasmids variants containing the above-mentioned genes may confer higher virulence for its host strain. Also, the chromosomal genes identified here belong to 2 key pathogenicity islands commonly found in isolates belonging to the B2 phylogenetic group (Clermont et al., 2001; Kariyawasam et al., 2006). Therefore, the “mix-and-match” of these chromosomal and plasmidic traits may also confer enhanced virulence to a strain, although this needs to be confirmed experimentally.

**Table 5:** Distribution of known avian pathogenic *E. coli* virulence and fitness factors (%) among *E. coli* populations categorized using APECTyper.

	Non-APEC (*n* = 99)	Non–high-risk APEC (*n* = 268)	High-risk APEC (*n* = 210)	Chi-squared *P* value (non–high-risk APEC vs. high-risk APEC)
*aatA*	34.3	28.0	9.5	<0.01
*aec35–37*	0.0	2.2	0.0	0.03
*cbi*	4.0	24.3	11.9	<0.01
*cma*	4.0	35.1	20.5	<0.01
*cmi*	11.1	38.4	19.0	<0.01
*cvaB*	12.1	68.3	78.6	0.01
*eitA–D*	7.1–9.1	46.6–47.0	60.0–62.9	<0.01
*etsA*	9.1	90.3	83.8	0.03
*etsB*	9.1	92.2	85.2	0.02
*etsC*	23.2	91.8	82.4	<0.01
*fyuA*	19.2	57.8	82.4	<0.01
*hlyF*	7.1	100.0	100.0	0.99
*ibeA*	10.1	11.9	61.0	<0.01
*iroN*	23.2	91.0	83.8	0.02
*irp2*	18.2	56.7	80.5	<0.01
*iss*	52.5	95.5	99.5	<0.01
*iucA–D*	13.1–14.1	74.3–78.0	86.7–89.5	<0.01
*iutA*	15.2	76.1	87.1	<0.01
*ompTp*	3.0	100.0	100.0	0.99
*papC*	25.3	18.7	50.0	<0.01
*sitA–D*	37.4	94.4–96.6	88.6–89.0	<0.01
*tia*	32.3	19.4	29.5	<0.01
*tsh*	3.0	49.3	63.8	<0.01
*vat*	21.2	32.1	21.9	0.01

**Figure 2:**
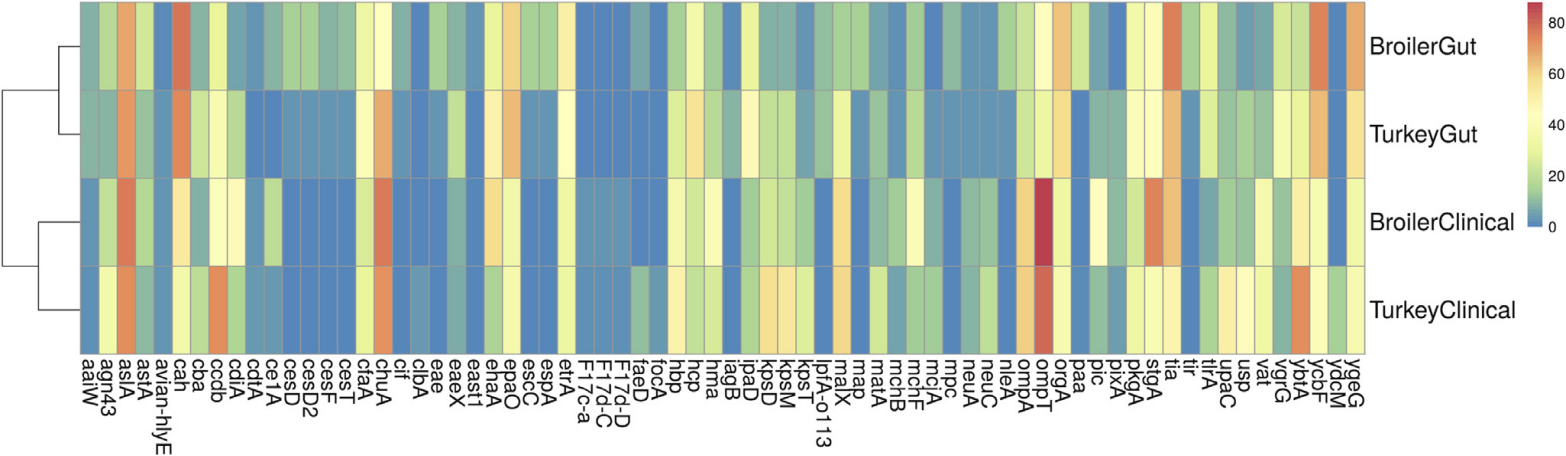
Heatmap depicting the prevalence of selected *E. coli* virulence and fitness factors across clinical and gastrointestinal isolates from broilers and turkeys. Colors represent percent presence of a gene within a source group.

Isolates were next screened for general *E. coli* virulence and fitness factors using the *E. coli* virulence database in VirulenceFinder, which searches for >2,701 gene alleles (Supplementary Data and Figure 2). Again, no genes were identified that were clearly defining of clinical vs. gastrointestinal isolates, for either broiler or turkey isolates. Several genes were found more frequently in broiler clinical isolates compared to broiler gastrointestinal isolates, including *ybtA*, another gene of the *Yersinia* high pathogenicity island (Geoffroy et al., 2000); *malX*, a maltose- and glucose-specific component IIa of a phosphoenolpyruvate-dependent phosphotransferase system (Ostblom et al., 2011); *hbp*, a hemoglobin protease autotransporter (Otto et al., 1998); *kpsD, kpsM*, and *kpsT*, bacterial capsular polysaccharide determinants (Russo et al., 1998); and *hma*, a heme acquisition receptor (Hagan and Mobley, 2009). Also, some genes were identified more frequently in broiler gastrointestinal isolates compared to broiler clinical isolates. For example, *cah* was found in 73% to 77% of gastrointestinal isolates and 36% to 43% of clinical isolates. This gene has been shown to be implicated with enhanced colonization and adherence properties in Shiga toxin-producing *E. coli* (Carter et al., 2018). Similarly, genes of type III secretion system 2 (*epaO, etrA*, and *ygeG*) were found more frequently in gastrointestinal vs. clinical isolates (Fox et al., 2020). Comparing broiler and turkey clinical isolates, broiler isolates had higher prevalence of *stgA* (74% vs. 40%), a marker of the *stg* fimbrial operon previously shown to enhance APEC colonization in chickens (Lymberopoulos et al., 2006); *cdiA* (47% vs. 17%), encoding a contact-dependent inhibition protein (Beck et al., 2016); *mchF* (40% vs. 14%), a component of the microcin H47 system (Azpiroz et al., 2001); and *pic* (43% vs. 11%), encoding a serine protease autotransporter (Abreu et al., 2016). Turkey clinical isolates had higher prevalence of *ccdB* (73% vs. 41%), encoding the toxin component of a toxin-antitoxin system (Bernard and Couturier, 1992); *ybtA* (72% vs. 52%), *kpsD* (57% vs. 24%) and *kpsM* (54% vs. 21%), *upaC* (51% vs. 14%), encoding an autotransporter (Allsopp et al., 2012); and *usp* (45% vs. 12%), encoding a genotoxin (Nipic et al., 2013). Many of the turkey-associated genes have been previously associated with *E. coli* phylogenetic group B2 (Clermont et al., 2001), which is in line with the higher proportion of turkey clinical isolates belonging to this group.

Another noteworthy finding was that some gastrointestinal isolates contained genes linked to enteropathogenic *E. coli*. These were found uniformly at a prevalence of approximately 14% in broiler isolates and 3% in turkey isolates, but were absent from broiler and turkey clinical isolates. Genes identified included *cesD*, a T3SS chaperone from the LEE pathogenicity island (Deng et al., 2004); *eae*, encoding for intimin on LEE; *escC*, encoding a T3SS secretin on LEE (Tseytin et al., 2018); *espA*, encoding a secreted signal transduction protein on LEE (Kenny et al., 1996); *map*, encoding a multi-function mitochondrial-associated protein on LEE (Nguyen et al., 2015b); *nleA*, encoding an EPEC effector protein (Nguyen et al., 2015b); and *tir*, encoding the intimin receptor (Deng et al., 2004). These isolates were devoid of the EAF plasmid gene markers in the virulence factor database, which would classify them as atypical EPEC (Hernandes et al., 2009). No toxin-encoding genes were found in the isolates linked to Enterohemorrhagic *E. coli* or Enterotoxigenic *E. coli*. Atypical EPEC (**aEPEC**) have been reported at relatively low frequency in broiler production and meat samples across the world but, to our knowledge, have not been reported in US broiler or turkey production (Farooq et al., 2009; Tanabe, 2013; Alonso et al., 2016; Adorjan et al., 2020; Zarei et al., 2021). These results illustrate that aEPEC do inhabit the gastrointestinal tracts of poultry in the United States, and could represent a possible threat to human health. However, their absence from clinical isolates suggests that they pose little threat to cause colibacillosis.

### Resistance Genes

Acquired antimicrobial resistance genes were scanned among isolates using ResFinder, and analyzed by source population (Table 6). Among broiler clinical isolates genes conferring resistance toward aminoglycosides (*aac(3)-VIa_2* and *ant(3″)-Ia_1*), sulfonamides (*sul1)*, and tetracyclines (*tet(A)*) were the dominant alleles identified. While genes conferring resistance toward the same classes of antimicrobials were identified among turkey clinical isolates, allele distributions were different. For example, turkey clinical isolates had a significantly lower prevalence of *aac(3)-VIa_2, ant(3″)-Ia_1*, and *sul1;* and significantly higher prevalence of *aph(3″)-Ib_2, aph(3′)-Ia_1, aph(6)-Id_1, sul2*, and *tet(B)*. Broiler gastrointestinal isolates had comparable resistance gene prevalence to broiler clinical isolates. In contrast, several resistance genes were of significantly higher prevalence in turkey gastrointestinal isolates compared to turkey clinical isolates, including *aac(3)-VIa_2, ant(3″)-Ia_1, aph(6)-Id_1, sul1*, and *sul2*. Notably, no acquired genes were identified which confer carbapenem or colistin resistance, and extended spectrum beta lactamase gene prevalence was relatively low across isolate populations.

**Table 6:** Distribution of dominant resistance gene alleles (%) by isolate source.

	Broiler clinical (*n* = 58)	Broiler gastrointestinal (*n* = 88)	Turkey clinical (*n* = 397)	Turkey gastrointestinal (*n* = 34)	*P* value
*aac(3)-IId_1*	8.9	6.7	12.1	14.7	0.41
*aac(3)-IVa_1*	3.6	2.2	10.4	14.7	NA
*aac(3)-VIa_2*	55.4^a^	41.1^a^	15.9^b^	41.2^a^	<0.01
*ant(3″)-Ia_1*	53.6^a^	45.6^a^	23^b^	44.1^a^	<0.01
*aph(3″)-Ib_2*	8.9^b^	17.8^ab^	26^a^	26.5^a^	0.02
*aph(3″)-Ib_5*	14.3^bc^	5.6^c^	22.2^ab^	32.4^a^	<0.01
*aph(3′)-Ia_1*	8.9^b^	5.6^b^	36.1^a^	32.4^a^	<0.01
*aph(4)-Ia_1*	3.6	2.2	10.6	14.7	NA
*aph(6)-Id_1*	23.2^c^	24.4^c^	44.9^b^	67.6^a^	<0.01
*bla*_CMY-2_1_	7.1	2.2	4.5	0	NA
*bla*_TEM-1B_1_	17.9^bc^	8.9^c^	29.8^ab^	44.1^a^	<0.01
*bla*_TEM-1C_1_	3.6	0	0.8	0	0.13
*dfrA1_10*	7.1	1.1	0	0	NA
*dfrA1_8*	7.1	2.2	1.5	0	NA
*fosA7_1*	3.6	2.2	0	0	0.66
*sul1_5*	51.8^a^	36.7^ab^	27.8^b^	47.1^a^	<0.01
*sul2_2*	26.8^c^	18.9^c^	43.4^b^	61.8^a^	<0.01
*tet(A)_6*	39.3	28.9	30	50	0.06
*tet(B)_2*	16.1^b^	16.7^b^	42.4^a^	58.8^a^	<0.01

NA = Test not performed because expected frequencies violated assumptions

a-c Indicate differences between groups based on statistical tests (P < 0.05).

### Plasmids

Plasmid replicons were screened using PlasmidFinder across the 4 source populations (Table 7). The most commonly occurring plasmid replicons across all populations included IncFIB(AP001918), Col(MG828), IncFIC(FII), and IncI1(Alpha). The IncFIB(AP001918) replicon was found significantly more often (P < 0.05) in clinical vs. gastrointestinal isolates, and significantly more often (*P* < 0.05) in broiler clinical vs. turkey clinical isolates. Similarly, the IncFIC(FII) replicon was found significantly more often in clinical vs. gastrointestinal isolates. This is likely reflective of the presence of the APEC plasmid in clinical populations, which frequently carry IncFIB or IncFIC plasmid replicons (Johnson et al., 2007). The Col(MG828) replicon was comparably prevalent in broiler clinical vs. gastrointestinal isolates, but was found at a significantly higher prevalence in turkey isolates. Furthermore, this replicon was found significantly more often in turkey gastrointestinal vs. turkey clinical isolates. This plasmid type is a small, nonconjugative plasmid (Kondratyeva et al., 2020). The IncI1(Alpha) plasmid replicon did not differ significantly between populations. The pO111 plasmid replicon was found significantly more frequently in gastrointestinal vs. clinical isolates. This plasmid has been correlated with isolates belonging to Enteroaggregative *E. coli* and diffusely adherent *E. coli* pathotypes (Montealegre et al., 2020), although markers of those pathotypes were not detected in this study.

**Table 7:** Distribution of plasmid replicons (%) by isolate source.

	Broiler clinical (*n* = 58)	Broiler gastrointestinal (*n* = 88)	Turkey clinical (*n* = 397)	Turkey gastrointestinal (*n* = 34)	*P* value
Col(MG828)	31.0^c^	37.5^c^	56.2^b^	73.5^a^	<0.01
Col(pHAD28)	8.62^b^	31.8^a^	7.3^b^	29.4^a^	<0.01
Col156	19	14.8	27.5	20.6	0.05
ColpVC	12.1^b^	11.4^b^	24.2^a^	0.0^c^	<0.01
IncFIB (AP001918)	100^a^	68.2^bc^	90.2^b^	61.8^c^	<0.01
IncFIB(K)	0.0^b^	1.1^b^	0.5^b^	23.5^a^	<0.01
IncFIC(FII)	56.9^a^	39.8^b^	64.2^a^	41.2^b^	<0.01
IncFII(29)	0^b^	8.0^a^	1.5^b^	5.9^ab^	<0.01
IncFII (pHN7A8)	0^b^	5.7^ab^	11.1^a^	5.9^ab^	0.02
IncFII	10.3	2.3	7.3	11.8	0.16
IncHI1A	1.7^b^	1.1^b^	0.8^b^	11.8^a^	<0.01
IncHI2A	6.9^bc^	3.4^c^	13.1^ab^	17.6^a^	0.03
IncHI2	6.9^bc^	3.41^c^	13.9^ab^	17.6^a^	0.02
IncI1-I (Alpha)	32.8	29.5	33.5	41.2	0.68
IncX1	12.1^b^	1.1^c^	26.7^a^	26.5^a^	<0.01
p0111	6.9^c^	19.3^b^	5.54^c^	35.3^a^	<0.01

Replicons shown are those present in at least 10% of any population.

a-c Indicate differences between groups based on statistical tests (P < 0.05).

### Overall Genetic Relationships Between Isolate Populations

**Figure 3:**
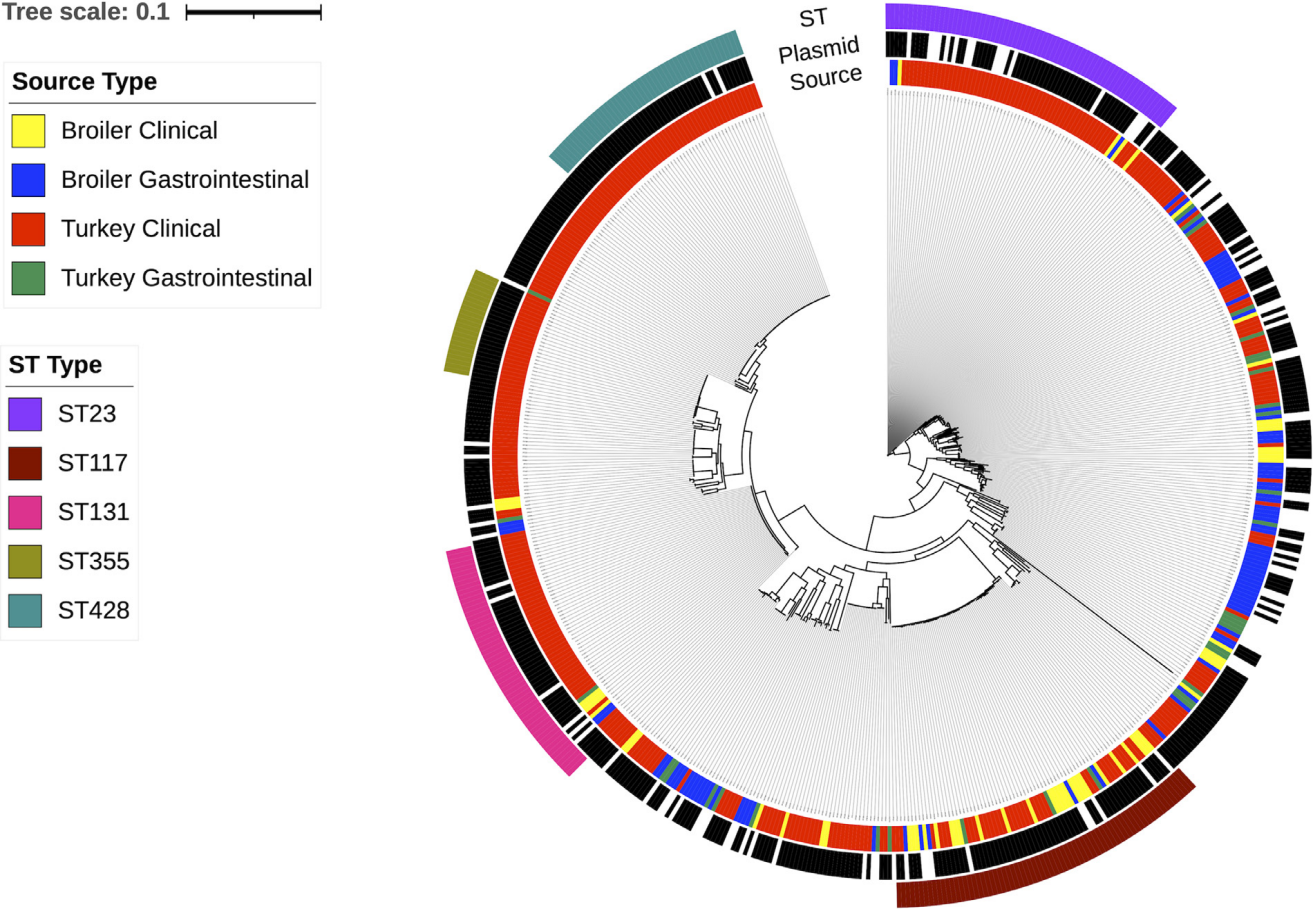
Phylogenetic tree depicting core SNP-level genomic relationships between isolates in this study. The inner ring depicts source type; the middle ring depicts presence (black) or absence (white) of the APEC plasmid based on possession on *hlyF* and *ompTp*; the outer ring depicts high-risk STs.

A core SNP-based phylogenetic tree was constructed to illustrate similarities and differences between isolates (Figure 3). High-risk STs differed by their sources. For example, ST355 and ST428 were exclusively turkey clinical isolates. ST23 and ST131 were dominated by turkey clinical isolates, but both STs contained representative isolates from every source type. In contrast, ST117 was more diverse and contained a much higher proportion of broiler clinical isolates, and also contained numerous gastrointestinal isolates. As a whole, though, gastrointestinal isolates generally fell outside of these dominant STs and were scattered throughout the phylogenetic tree. This confirms the diversity of clones within the avian gut and the relatively infrequent occurrence of high-risk clones in the gut.

We then examined SNP level differences within the high-risk STs where multiple source types were represented. This demonstrated 2 key points: 1) there were multiple examples of clonal relatedness between clinical and gastrointestinal isolates, and 2) there were multiple examples of clonal relatedness between broiler and turkey isolates. For example, the range of SNP differences within ST23 isolates was 0-184, and most of these were turkey clinical isolates. A broiler gastrointestinal ST23 isolate differed by only 7 SNPs from multiple turkey clinical isolates. ST117 isolates were much more diverse, as expected, and ranged from 0 to 939 SNP differences. However, many isolates were identified which can be considered clonal. For example, turkey clinical and broiler gastrointestinal ST117 isolates were found differing by only 7 SNPs. Broiler gastrointestinal and broiler clinical ST117 isolates were found differing by only 3 SNPs. ST131 isolates differed by 0 to 264 SNPs. Here, we identified much more homogeneity within turkey clinical isolates and some distinction between turkey and broiler isolates, and between clinical and gastrointestinal isolates. Still, examples of clonal relatedness were also identified within ST131, including turkey clinical and turkey gastrointestinal isolates differing by 7 SNPs; a turkey clinical isolate and broiler clinical isolate differing by 11 SNPs; and a broiler clinical isolate and broiler gastrointestinal isolate differing by 3 SNPs. Such clonal relatedness between isolates from differing source populations is remarkable, given that these samples were collected across time and space from different bird types and different flocks. This further reinforces the idea that common high-risk clones exist, which are successful in poultry production, irrespective of bird type or geography, and they have low-level presence in the gastrointestinal tract.

## CONCLUSIONS

This study was conducted to gain better understanding of the relationships between gastrointestinal isolates and clinical isolates from broilers and turkeys, and to determine if APECTyper using the concept of high-risk clonal groups is useful in both broilers and turkeys. Our results indicate that, while the distribution of isolates across these high-risk groups certainly differs between broiler and turkeys, it does capture the dominant and high-risk clones in both populations. A better understanding of the ecology of *E. coli* across the gastrointestinal tract, other anatomical sites in the bird, and within the production environment is greatly needed if we are to elucidate where and why these clones are gaining success.

## Appendix Supplementary materials

Image, application 1

## Footnotes

Supplementary material associated with this article can be found in the online version at doi:10.1016/j.psj.2023.102712.
